# Amputations in the Pennsylvania Hospital

**Published:** 1870-12

**Authors:** 


AMPUTATIONS IN THE PENNSYLVANIA HOSPITAL.—Dr. T. G. Morton has prepared tables giving an account of all cases of amputation in the hospital named, during the decade ending January 1, 1870, with a statement of the mortality from this operation during forty years.

During the ten years there were performed 321 major amputations, upon 311 patients. Of these 227 were cured, 83 died, and 1 removed.

| | | | | | | |
|---|---|---|---|---|---|---|
| Of 30 | foot and ankle amputations, | 22 | were | cured, | 8 | died. |
| " 29 | thigh " | 17 | " | " | 12 | " |
| " 5 | hip-joint " | 2 | " | " | 3 | " |
| " 22 | knee-joint " | 12 | " | " | 8 | " |
| " 102 | leg " | 70 | " | " | 32 | " |
| " 10 | shoulder-joint " | 7 | " | " | 3 | " |
| " 50 | arm " | 34 | " | " | 16 | " |
| " 2 | elbow " | 2 | " | " | 0 | " |
| " 48 | fore arm* " | 42 | " | " | 5 | " |

*1 Removed by request.

Of 23 wrist and part hand amputations all were cured.

Of the 321 amputations, 239 were primary; of these 176 were cured, 63 died. 22 were secondary operations, of which 11 were cured, and 11 died; 60 were amputations for chronic diseases, of which 51 were cured, and 9 died.

Of 61 amputations at the joints, 44 were cured, and 17 died. Out of the 134 amputations at the upper extremities, 111 were cured, and 23 died. Of 187 amputations of the lower extremities, 124 were cured, and 63 died.

114 patients were under 20 years of age; of these 98 were cured, and 16 died. 84 patients were between 20 and 30; of these 63 were cured, and 21 died. 65 patients were between 30 and 40; of these 45 were cured, and 20 died. 25 patients were between 40 and 50; of these 16 were cured, and 9 died. 23 patients were above 50; of these 7 were cured. and 16 died.

During the forty years ending with January last, 749 amputations were performed on 735 patients; 548 were cured, 186 died, and 1 removed. 500 were primary operations, performed during the first twenty-four hours after injury, of which 117 died; 105 were secondary, of which 42 died; 144 were for chronic diseases, of which 27 died.

232 of the patients were under 20 years of age, of whom 206 were cured, and 26 died. 217 were between 20 and 30, of whom 164 were cured, and 53 died. 152 were between 30 and 40, of whom 105 were cured, and 47 died. 87 were between 40 and 50, of whom 56 were cured, and 31 died. 44 were upwards of 50, of whom 23 were cured, and 21 died.

Ether has been almost invariably used as the anæsthetic, and "no death has, I believe, ever occurred in the hospital from its use." At times, ether has been combined with chloroform, and one sudden death occurred from this mixture, during an operation for necrosis. —*American Journal Medical Science.*

